# A Large Pilar Sheath Acanthoma of the Cheek Successfully Treated With Surgical Excision

**DOI:** 10.7759/cureus.62364

**Published:** 2024-06-14

**Authors:** Yelena Dokic, Maria S Bloomquist, Hafeez Diwan, Omid Jalali, Jennifer S Ranario

**Affiliations:** 1 Dermatology, Baylor College of Medicine, Houston, USA; 2 Pathology and Immunology, Baylor College of Medicine, Houston, USA

**Keywords:** follicular hamartoma, facial cyst, cyst, surgical excision, pilar sheath acanthoma

## Abstract

This case report describes an atypically large pilar sheath acanthoma (PSA) presenting on a patient's cheek. Due to the bothersome nature of the lesion, the patient underwent surgical excision, with subsequent histopathological analysis confirming the diagnosis of an unusually large PSA. In addition to a definitive diagnosis, surgical excision provided symptomatic relief for the patient.

## Introduction

In this case report, we present a unique instance of a pilar sheath acanthoma (PSA). PSAs are typically small, benign growths of the hair follicle presenting as a single bump with a central opening. They most commonly appear on the upper lip in middle-aged or older adults, but can occur anywhere on the head and neck. Originating from the hair follicle's outer root sheath, these lesions are usually asymptomatic and require no treatment [1,2]. However, surgical excision can be performed for symptomatic relief, infection, irritation, or cosmetic reasons, particularly when the PSA is larger than usual. The standard size of a PSA falls between 0.5 and 1.0 cm [3]. In this case, our patient presented with an unusually large PSA measuring 1.5 cm in diameter. This inflamed and draining lesion prompted the patient to seek surgical removal for definitive diagnosis and symptomatic relief.

## Case presentation

**Figure 1:**
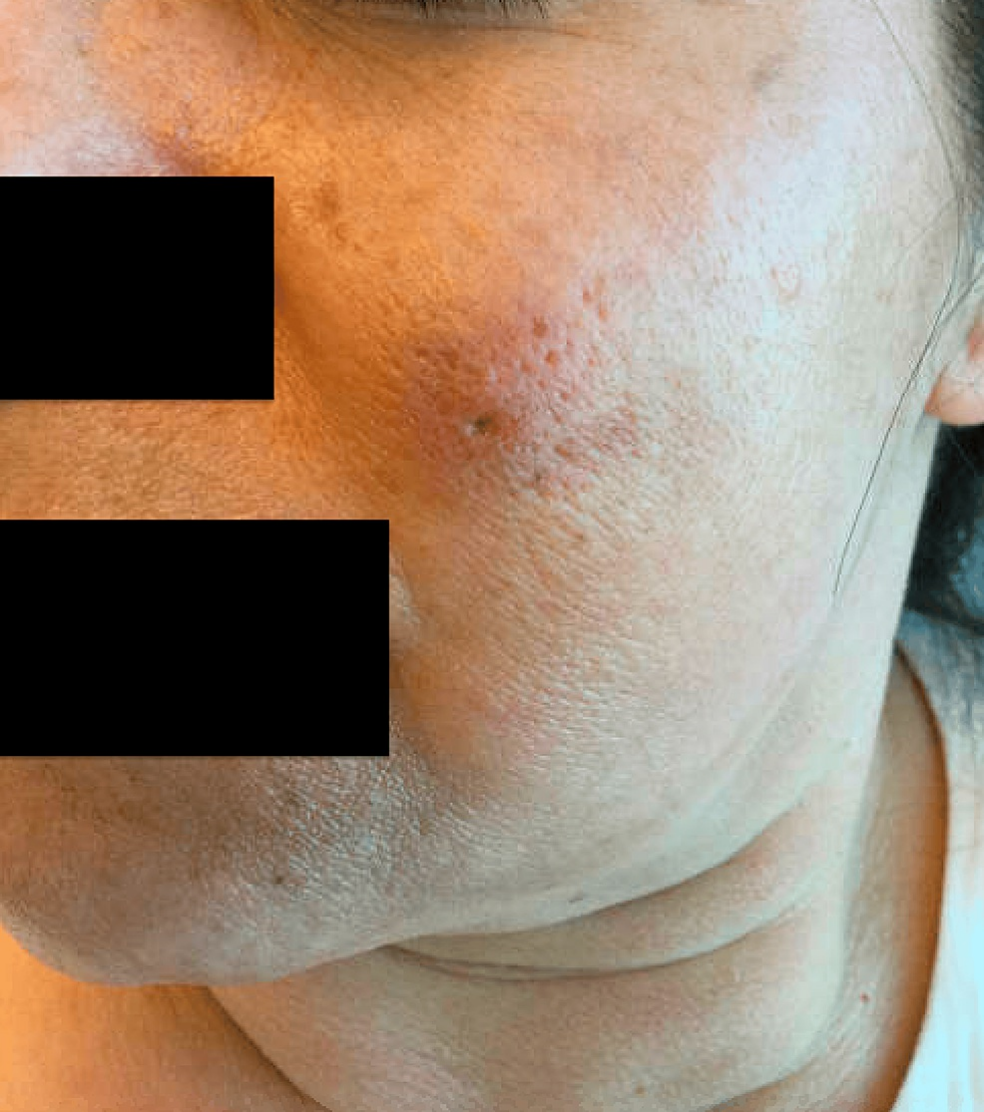
Subcutaneous nodule with central punctum on the left cheek.

**Figure 2:**
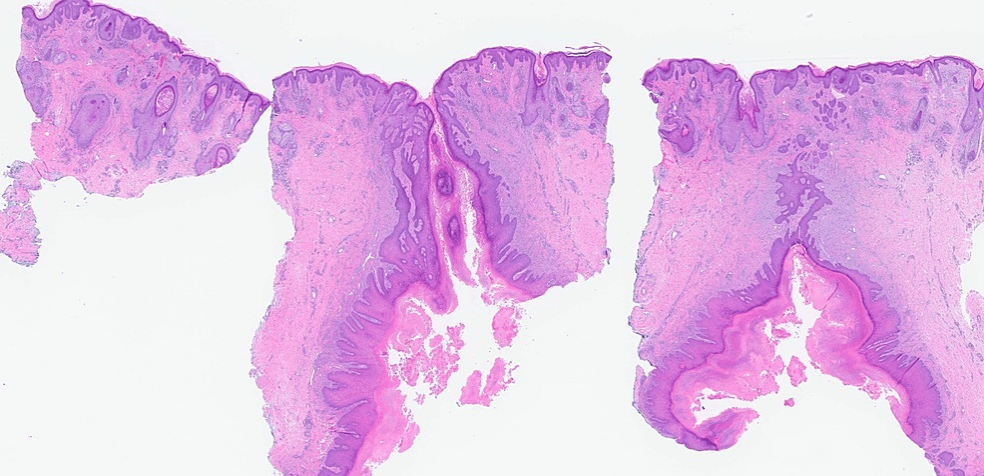
H&E, original magnification x10.

**Figure 3:**
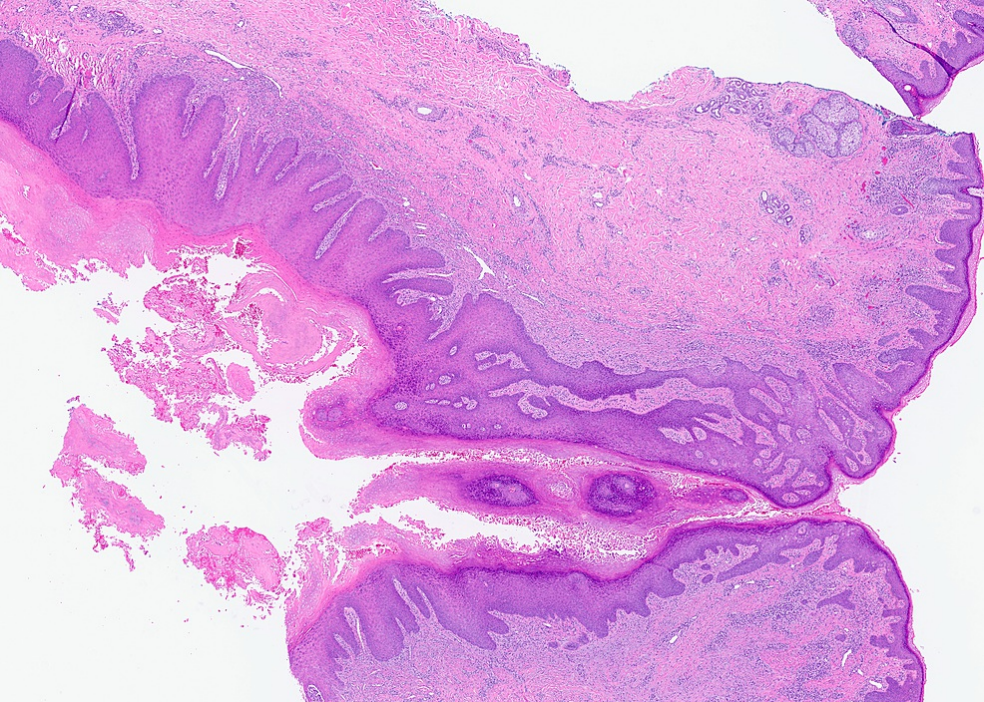
H&E, original magnification x20.

**Figure 4:**
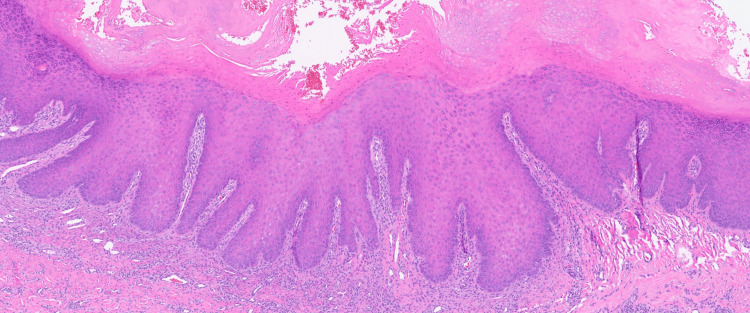
H&E, original magnification x50.

A 55-year-old healthy woman presented with a 1.5-cm erythematous subcutaneous nodule on her left cheek that had been present for six months. Over the past three months, the nodule had worsened, becoming larger, more tender, and draining. The patient had undergone multiple courses of oral antibiotics, including doxycycline, clindamycin, and amoxicillin, with no improvement. Physical examination revealed a solitary erythematous nodule with a central punctum on the left cheek (Figure 1). Surgical excision was performed for both diagnostic and therapeutic purposes. Histopathological examination demonstrated acanthotic lobules attached to a dilated follicular pore (Figures 2-3). The infundibulum was dilated with a central pore connecting to the epidermis, and there were acanthotic extensions of the infundibulum lacking hair shafts. These histological features were diagnostic of a PSA (Figure 4).

## Discussion

A PSA is a benign, follicular hamartoma that typically presents as a small, solitary papule with a central punctum. It is most commonly located on the upper lip of middle-aged and elderly adults, but can also be found anywhere on the head or neck [1]. This benign neoplasm arises from the outer root sheath of the hair follicle and is thus derived from follicular epithelium [2]. PSA is usually asymptomatic and does not require treatment. However, if the lesion is enlarging, bothersome, irritated, or infected, it may be surgically excised [3]. It also can be surgically excised for cosmetic purposes. Typically, PSAs are between 0.5 and 1.0 cm [4]. Our patient presented with a notably large PSA, measuring 1.5 cm in diameter. The lesion was inflamed and draining, which led our patient to seek removal.

PSA is a relatively rare lesion, with an estimated incidence of 0.1-0.2% [5]. It is most common in adults over the age of 50, with a slight male predominance. Clinically, PSA appears as a cyst versus other benign neoplasms, with a central punctum. However, it is sometimes biopsied or surgically excised to rule out a malignant process. Histopathologically, while PSAs are not true cysts, some lesions may have cystic architecture.

The histopathologic features of PSAs are characteristic and can be used to make the diagnosis. The lesion is composed of a proliferation of squamous epithelium that forms lobules in the dermis surrounding a central cystic space. The lining cells of the lobules may have a granular layer, similar to a follicular infundibular cyst, or they may have an attenuated granular layer, similar to a trichilemmal cyst [1,6]. The lobules are composed of bland keratinocytes, which are surrounded in areas by an eosinophilic basement membrane. There are often areas of clear cells within the lobules.

Histologic mimics of a PSA include a tumor of the follicular infundibulum, trichofolliculoma, inverted follicular keratosis, and dilated pores of Winer.

Tumor of follicular infundibulum is a rare benign adnexal neoplasm. Clinically, this lesion appears as a thin, hypopigmented papule or macule. Occasionally, several lesions may be present in one patient. Histologic features include pale keratinocytes forming thin, reticular anastomosing branches that run parallel to the epidermis [1]. These keratinocytes have continuity with both the surface of the epidermis, and the surrounding follicular structures [7].

A trichofolliculoma clinically appears as a papule or nodule on the face, scalp, or upper trunk, with vellus hairs emerging from a centrally dilated pore. Histopathology reveals a central cystic space with central orthokeratin and infundibular cornification [8]. Secondary and tertiary hair follicles emanate from the walls of the central cystic space, and a fibrous stroma envelops the lesion [1]. The trichofolliculoma has fully formed follicular structures, while the PSA only has the isthmus and outer sheath. Additionally, the stroma in the trichofolliculoma is more fibrous compared to the PSA, in which the stroma is minimal to absent.

Inverted follicular keratosis presents as a firm, tan, white, or pink papule on the face and neck of middle-aged or older adults [9]. It has a predilection for the cheek and upper lip. Histologically, there is an endophytic proliferation of eosinophilic keratinocytes with basaloid or squamous differentiation, commonly with squamous eddies [1].

A dilated pore of Winer presents clinically as a solitary, dilated comedo that is filled with firm keratin. It occurs primarily on the face of adults [10]. Histologically, keratinaceous debris can be seen filling a dilated follicular opening, with a lining of squamous epithelium including a granular layer. The lining is acanthotic and exhibits finger-like projections protruding into the surrounding dermis [1].

## Conclusions

We report this case to describe an unusual presentation of a large PSA on the cheek of a patient that was definitively diagnosed by dermatopathology after surgical excision. This case serves as a reminder that, while follicular infundibular cysts are the most common type of cyst, more rare neoplasms such as PSA can also arise. Therefore, it is important to keep a wide differential diagnosis for subcutaneous nodules in patients. The value of surgical excision in this case was twofold: it provided relief for the patient, as the lesion was bothersome, and it achieved a definitive dermatopathological diagnosis, confirming the benign nature of the lesion.
